# MULTIMODAL IMAGING OF BILATERAL IDIOPATHIC MULTIFOCAL RETINAL PIGMENT EPITHELIAL DETACHMENTS IN YOUNG PATIENTS

**DOI:** 10.1097/ICB.0000000000001274

**Published:** 2023-10-23

**Authors:** Sulaiman Alhumaid, Noy Ashkenazy, Julia L. Hudson, Audina M. Berrocal, Harry W. Flynn

**Affiliations:** * Department of Ophthalmology, Bascom Palmer Eye Institute, University of Miami Miller School of Medicine, Miami, Florida; and; † Retina Macula Specialists of Miami, North Miami Beach, Florida.

**Keywords:** PEDs, multifocal PEDs, multimodal imaging, optical coherence tomography, PEDs in young patients

## Abstract

**Purpose:**

The aim of this study was to describe two cases of multiple epithelial detachments (pigment epithelial detachments [PEDs]) occurring in otherwise young, healthy patients.

**Methods:**

The medical and imaging records of two cases were reviewed retrospectively.

**Results:**

Multiple serous nonvascularized PEDs were present in two young, healthy patients who underwent multimodal imaging. The first patient, a 38-year-old woman, was incidentally found to have a visual acuity of 20/20 in both eyes and multiple bilateral PEDs. Imaging and a systemic work-up was negative for secondary causes, and at 36 months, vision remained stable although some of the PEDs had coalesced. The second patient, a 32-year-old woman, presented with distortion and perceived scotomas in both eyes progressive over the course of 2 years. The visual acuity was 20/20, and multimodal imaging confirmed the presence of bilateral PEDs.

**Conclusion:**

Bilateral idiopathic multifocal retinal PEDs in otherwise healthy young adults is a rare condition. Longer term follow-up is needed to evaluate secondary complications and visual outcomes.

## Abstract 2

We present the clinical and imaging features of two otherwise young, healthy patients with multiple serous, nonvascularized pigment epithelial detachments. This rare interesting entity was previously described by Gass in 2005. The current article uses multimodal imaging to provide further documentation of the retinal features.

A bilateral rare condition of multifocal bilateral idiopathic retinal pigment epithelial detachments occurring in otherwise healthy adults was described by Gass in 2005. At that time, the condition was considered to be a variant of idiopathic central serous chorioretinopathy.^1^ Pigment epithelial detachments may be features of different chorioretinal diseases, more commonly seen in association with age-related macular degeneration,^2^ polypoidal choroidal vasculopathy,^3^ central serous chorioretinopathy–like changes in pregnancy,^4^ and systemic corticosteroid treatment.^5^ It may also be observed in patients with malignant hypertension, hypercoagulable state, and increased viscosity and patients on hemodialysis.^6^ It may occur in patients with inflammatory conditions such as Vogt–Koyanagi–Harada disease and sarcoidosis.^7^ Pigment epithelial detachments (PEDs) have been described in patients with non-Hodgkin lymphoma^8^ and leukemia.^9^ We herein report the clinical features and multimodal imaging findings of bilateral multiple retinal PEDs in two young, healthy women.

## Case Reports

### Case 1

A 38-year-old pregnant woman with no visual complaints was referred to the Bascom Palmer Eye Institute by an outside eye health provider after being diagnosed with bilateral multifocal PEDs during a screening for Plaquenil treatment. Her medical history was significant for mixed connective tissue disease with positive antinuclear antibody and rheumatoid factor, polycystic ovary syndrome, and hypothyroidism. The patient had a positive dengue disease test a year before presentation. The patient presented during the twentieth week of gestation. Her family history was unremarkable.

**Fig. 1.**
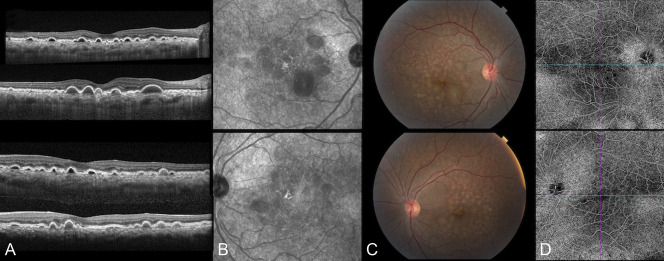
Top panel: right eye and bottom panel: left eye. **A.** Optical coherence tomography (Heidelberg Spectralis, Heidelberg Engineering, Germany) demonstrated multiple small serous PEDs at presentation (top) which coalesced into larger, broadened PEDs at 56 months of follow-up [bottom]. Similar findings are shown in the left eye but to a lesser degree than the right eye. **B.** Near-infrared images (Heidelberg Spectralis, Heidelberg Engineering, Germany) illustrate the serous PEDs within the macula seen on OCT at 56 months follow-up. **C.** Color fundus photographs (Topcon Healthcare, NJ) at 56 months after the initial presentation show multiple punctate orange lesions, consistent with punctate epithelial detachments involving the posterior pole. **D.** Swept-source optical coherence tomography angiography (SS-OCTA—PLEX Elite 9000, Carl Zeiss Meditec, Dublin, CA) 12 × 12 scans were analyzed looking at the slab between the retinal pigment epithelium and Bruch membrane. *En face* structural slab shows the presence of PEDs without Type 1 choroidal neovascularization.

Her visual acuity on initial examination was 20/20 in both eyes with myopic correction. Amsler grid testing was negative for both eyes. Color vision was also normal bilaterally. Pupils were equal, round, and reactive to light with no afferent pupillary defect in either eye. The anterior segment examination was unremarkable. Intraocular pressures were 20 in the right eye and 21 in the left eye by Goldmann applanation tonometer. On dilated fundus examination, there were multiple serous PEDs in the macula extending beyond the vascular arcades in both eyes (Figure 1). There was no hemorrhage, exudate, drusen, or subretinal fluid. Optical coherence tomography (OCT) (Heidelberg Spectralis, Heidelberg Engineering, Germany) demonstrated multiple dome-shaped serous, nonvascularized retinal pigment epithelial detachments with no neurosensory detachment and without evidence of choroidal neovascularization on OCT angiography (Zeiss, Cirrus Angio PLEX) (Figure 1). Choroidal thickness, measured from the retinal pigment epithelium to the choroidal–scleral junction, was 380 *μ*m bilaterally.

Her antinuclear antibody was positive and consistent with her mixed connective disease, while her QuantiFERON-gold; reactive plasma reagin; protein electrophoresis; complete blood count with differential, free kappa, and lambda light chains; and chikungunya antibodies testing were not significant for any other systemic inflammatory disease or infectious and malignant causes.

Her prepregnancy OCTs were similar to her follow-up OCTs. Throughout her 56 months follow-up, she remained asymptomatic with no changes in her vision. Some of the PEDs have coalesced and become larger in size but without choroidal neovascular membrane (Figure 1).

### Case 2

A 32-year-old woman with a history of breast fibroadenomas, dysmenorrhea, liver hemangioma, and anxiety presented with complaints of 2 years of distortion and scotomas in the central visual field of both eyes. She was seen by an outside provider who diagnosed her with drusen and provided her with an amsler grid. She noticed increased distortion on amsler testing, which prompted her visit. On presentation to the Bascom Palmer Eye Institute, the uncorrected visual acuity was 20/20 in both eyes. Intraocular pressure was 20 mmHg in the right eye and 21 mmHg in the left eye by tonopen. Pupillary light reflexes were normal without an afference pupillary defect bilaterally. External and anterior segment examinations were normal. The dilated fundus examination of the macula in both eyes revealed scattered round, hypopigmented PEDs which extended just outside of the temporal arcades and nasal to the disc. The vessels and remainder of the periphery were normal bilaterally.

**Fig. 2.**
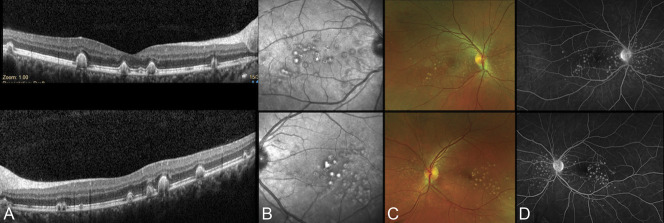
Top panel: right eye and bottom panel: left eye. **A.** Optical coherence tomography (Heidelberg Spectralis, Heidelberg Engineering, Germany) showing multiple retinal PEDs throughout the macula. There are numerous PEDs without associated subretinal fluid or choroidal neovascular membrane. **B.** Corresponding near-infrared images (Heidelberg Spectralis, Heidelberg Engineering, Germany) illustrate the punctate scattered configuration of these findings. **C.** Fundus photographs (Optos, United Kingdom) demonstrate yellow macula deposits with punctate areas within the macula. **D.** Corresponding fluorescein angiography revealed nonleaking punctate areas of hyperfluorescence in a staining pattern centered around the disc and macula.

The patient denied any hormone use such as systemic contraceptive medications or steroids. Multimodal imaging, including OCT (Heidelberg Spectralis, Heidelberg Engineering, Germany), revealed numerous serous, nonvascularized PEDs without associated subretinal fluid or choroidal neovascular membrane (Figure 2). Choroidal thickness was unable to be obtained. The corresponding fundus photographs and autofluorescence (Optos, United Kingdom) demonstrated yellow macula deposits with hypoautofluorescence. Optos fluorescein angiography and indocyanine green angiography revealed nonleaking punctate areas of hyperfluorescence in a staining pattern centered around the disc and macula (Figure 2). Panel-based genetic testing (Spark Therapeutics, Invitae Diagnostics) did not reveal any pathogenic mutations. The patient was observed without immediate intervention.

## Discussion

The clinical entity of bilateral multifocal idiopathic PEDs in young healthy individuals has been previously reported.^1,10^ Gass et al reported three patients with multiple PEDs that were otherwise healthy, one of which had enucleation for a vitreous hemorrhage secondary to a choroidal neovascular membrane, which was evaluated histologically. Goncu et al reported two cases with multiple PEDs in asymptomatic patients, one of which had subretinal pigment epithelial hemorrhage that regressed spontaneously. Both of these patients remained asymptomatic through the follow-up period.^10^ Other entities that can present with multiple PEDs include age-related macular degeneration^11^ and non-Hodgkin lymphoma.^8^ A recent entity was described as bilateral large colloid drusen in a young adult, presenting with solid appearing PEDs as well.^12^

Systemic inflammatory diseases such as Vogt–Koyanagi–Harada clinically presents with PEDs, exudative retinal detachment, choroidal thickening, anterior chamber and vitreous cells, and rarely choroidal neovascular membrane.^7^ Infectious causes including syphilis are uncommonly associated with PEDs. PEDs with exudative retinal detachments have been seen in patients with renal disease on dialysis.^6^

Pachychoroid should remain on the differential in a young patient with multiple PEDs. Pachychoroid pigment epitheliopathy is associated with PEDs in the setting of choroidal hyperpermeability on indocyanine green angiography and choroidal thickening on enhanced-depth imaging OCT.^13–15^ Choroidal thickness and indocyanine green in our cases were below the usual threshold for pachychoroid disease spectrum. Inherited macular dystrophy, such as Doyne macular dystrophy and malattia leventinese, was not suspected, based on an inconsistent clinical phenotype with absent drusen or drusenoid deposits in both cases.^16,17^

The clinical findings and follow-up course in our patient resembles those cases described by Gass et al ^1,4–6,8^ Longitudinal follow-up is needed to evaluate secondary complications and visual outcomes.

## Conclusion

The clinical entity of bilateral multifocal idiopathic retinal PEDs in young healthy individuals is documented on multimodal imaging. Longer follow-up will provide information on visual acuity outcomes and any secondary complications.
